# Association between mean ocular perfusion pressure and proliferative diabetic retinopathy in a northern Chinese population

**DOI:** 10.3389/fendo.2026.1921927

**Published:** 2026-10-23

**Authors:** Fei Gao, Han Bo, Emmanuel Eric Pazo, Liying Hu, Chen Chen, Yu Shi, Xiuqing Zhu, Xiaorong Li, Juping Liu

**Affiliations:** Tianjin Key Laboratory of Retinal Functions and Diseases, Tianjin Branch of National Clinical Research Center for Ocular Disease, Eye Institute and School of Optometry, Tianjin Medical University Eye Hospital, Tianjin, China

**Keywords:** blood pressure, diabetes mellitus, diabetic retinopathy, mean ocular perfusion pressure, pulse pressure

## Abstract

**Background:**

To evaluate the associations of mean ocular perfusion pressure (MOPP), pulse pressure (PP), and systolic blood pressure (SBP) with proliferative diabetic retinopathy (PDR) among patients with type 2 diabetes in a northern Chinese population.

**Methods:**

A retrospective cross-sectional study included 1,323 participants, comprising 1,119 patients from the Beichen Eye Study and 204 patients identified from electronic medical records at the Tianjin Medical University Eye Hospital. The participants were categorized into no diabetic retinopathy (NDR), non-proliferative diabetic retinopathy (NPDR), and PDR groups. SBP, diastolic blood pressure (DBP), and intraocular pressure (IOP) were measured. PP was defined as the difference between SBP and DBP. MOPP was calculated using the formula: MOPP = 2/3 (DBP + 1/3 PP) − IOP.

**Results:**

With PDR as the reference category, higher MOPP was associated with PDR relative to both NDR (OR, 0.92; 95% CI, 0.89–0.95) and NPDR (OR, 0.94; 95% CI, 0.91–0.98). SBP and PP showed similar patterns in their respective adjusted models and tertile analyses. Exploratory analyses showed evidence of nonlinear associations for PP (P for likelihood test = 0.046) and IOP (P for likelihood test < 0.001), but not for SBP or MOPP.

**Conclusions:**

Higher MOPP was associated with PDR relative to both NDR and NPDR, suggesting that MOPP may provide complementary hemodynamic information in relation to DR severity.

## 1 Introduction

Diabetes mellitus (DM) is a growing global health challenge, currently affecting 12.8% of adults in China (1). As one of the most severe ocular complications of DM, diabetic retinopathy (DR) remains a leading cause of blindness especially among the working-age population worldwide (2). It is estimated that DR affects roughly 22.3% of diabetic patients, with prevalence rates steadily increasing (3). Clinically, damage caused by DR starts as non-proliferative diabetic retinopathy (NPDR) and progresses to proliferative diabetic retinopathy (PDR) (4). A thorough understanding of the determinants of DR onset and progression is imperative, as effective management hinges on both preventing the disease and curtailing its advancement post-manifestation.

Each stage of DR is characterized by abnormalities in retinal blood flow, which is influenced by ocular perfusion pressure (5, 6). Mean ocular perfusion pressure (MOPP) is a calculated estimate of ocular perfusion status and has been associated with DR in previous cross-sectional and longitudinal studies, although the findings have not been entirely consistent (7–9). In this study, MOPP was calculated as MOPP = 2/3 (DBP + 1/3 PP) − IOP, where PP was defined as SBP minus DBP. Therefore, MOPP is principally influenced by SBP, DBP, and IOP. Blood pressure can be modified through the management of hypertension, whereas IOP may be modified when clinically indicated. However, because MOPP incorporates these interrelated parameters, clarifying the respective associations of MOPP and its component parameters with DR severity remains clinically relevant.

Hypertension has long been identified as a well-established risk factor for the development of PDR (10). Recent evidence from patients with newly diagnosed T2DM has shown that higher nighttime SBP was associated with retinal vascular caliber changes, an early marker of microvascular alteration. In that study, the association of nighttime SBP with retinal vascular caliber was stronger than that of hyperglycemia (11). Pulse pressure (PP), calculated by systolic blood pressure (SBP) minus diastolic blood pressure (DBP), has garnered growing attention for its pathologic significance in DR (12). A previous study reported that PP was more strongly associated than SBP with progression to severe DR (13). To date, most studies have examined the associations of MOPP, SBP, or PP with DR separately. Few investigations have directly compared these indicators within the same cohort. A clearer understanding of their respective associations with DR severity may have clinical relevance.

In this study, we investigated the associations of MOPP, PP, and SBP with DR severity in patients with T2DM in a northern Chinese population. Using PDR as the reference group, we assessed the associations of these parameters with NDR and NPDR to characterize their relationships across DR severity categories.

## 2 Materials and methods

### 2.1 Study design and population

This retrospective cross-sectional study included 1,119 participants from the Beichen Eye Study (BCES) and 204 additional patients with PDR from the electronic medical record database of Tianjin Medical University Eye Hospital. The detailed methodology of the BCES has been described previously (14). Briefly, from June 2020 to February 2022, 5,840 residents aged ≥ 50 years living in the Beichen District of Tianjin were enrolled in this epidemiological investigation using a multi-stage, stratified, cluster random sampling method. The ethics approval was obtained from the Tianjin Medical University Eye Hospital (TMUEH) Ethics Committee (2019ky-22) and this study was registered on the website of the Chinese clinical trial registry (ChiCTR2000032280). All participants signed the informed consent voluntarily. In consideration of the small sample of PDR in BCES, we expanded the sample size by including additional PDR cases from the electronic medical record database of TMUEH, covering the period from April 2000 to August 2024. Clinical data from 2,513 patients aged over 50 years old with T2DM who underwent pars plana vitrectomy for PDR were retrospectively retrieved from medical records. The inclusion criteria included gradable fundus photographs and the availability of data on blood pressure (BP) and MOPP. Based on the exclusion criteria, a total of 204 subjects were included in the final analysis. The use of data for this study was approved by the Institutional Review Board of Tianjin Medical University Eye Hospital (2024KY(L)-43). The board waived the requirement for informed consent given the retrospective design involving existing data from the medical record system. A detailed flowchart of participant selection is presented in **Figure 1**.

**Figure 1:**
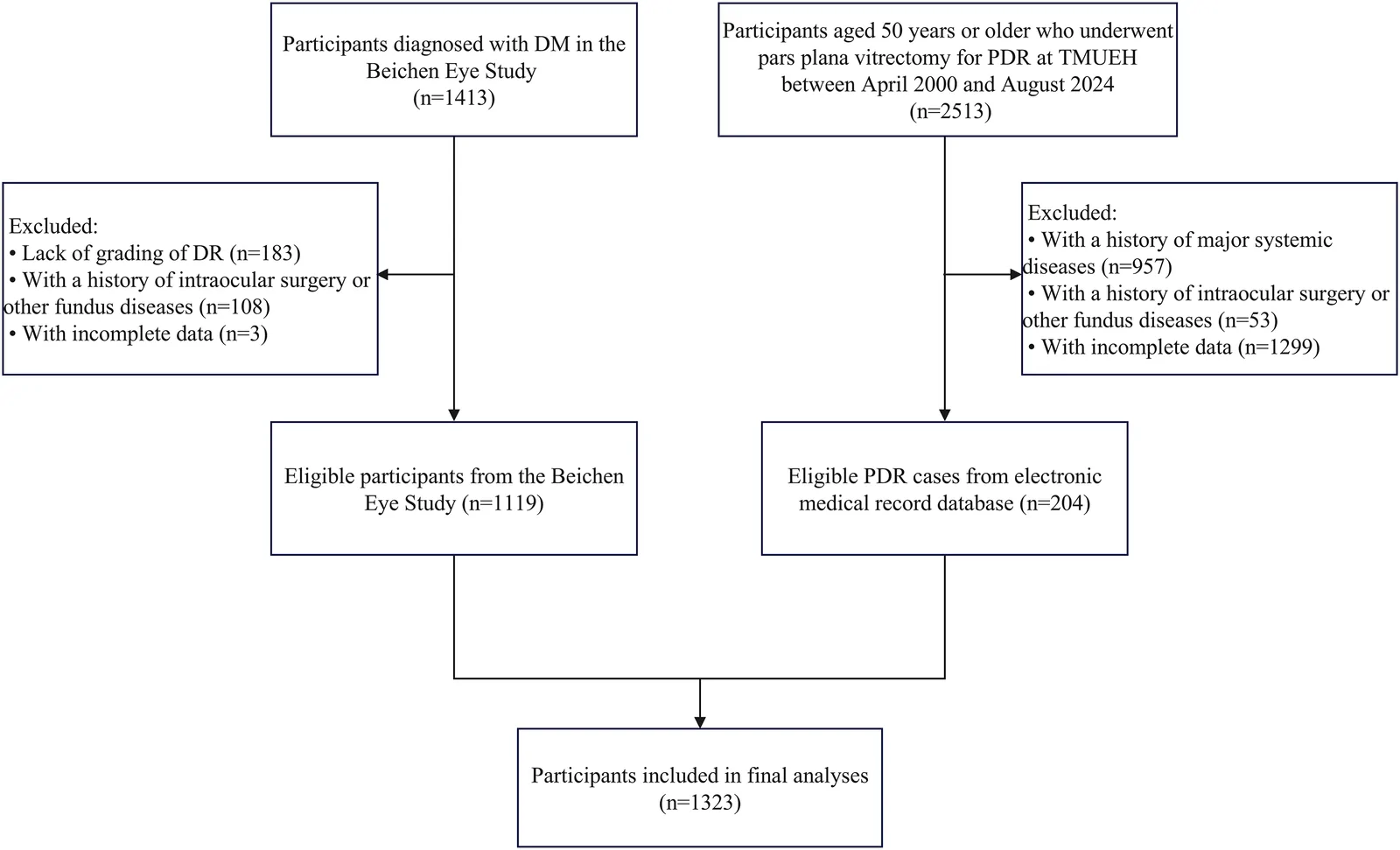
Flowchart of participant selection. DM, Diabetes mellitus; PDR, Proliferative diabetic retinopathy; TMUEH, Tianjin Medical University Eye Hospital.

### 2.2 Data collection

Demographic data and medical history, including age, sex, duration of DM and insulin usage were collected using a standardized questionnaire or by reviewing medical records. A comprehensive clinical eye examination, including visual acuity (VA), intraocular pressure (IOP), and color fundus photography, was performed on all participants. Blood samples were collected to assess blood glucose, hemoglobin A1c (HbA1c), total cholesterol (TC), triglycerides (TG), alanine transaminase (ALT), aspartate transaminase (AST), uric acid (UA) and creatinine (CRE) according to the standardized procedures of local accredited laboratories. After participants had rested in a seated position for 5 minutes, trained technicians measured SBP and DBP three times using an automatic electronic sphygmomanometer (OMRON, Japan). The mean of the three measurements was used in all analyses (15). IOP was measured three times with an AccuPen Handheld Applanation Tonometer (24–3000, Accutome, Inc., USA) and the averaged value was used for the statistical analysis. PP was computed as SBP minus DBP and MOPP was calculated by the following formula: MOPP = 2/3 (DBP + 1/3 PP) – IOP (16). In addition, we also calculated body mass index (BMI) and cerebrospinal fluid pressure (CSFP) using the following formulas: BMI = weight/height^2^ and CSFP = 0.44 × BMI + 0.16 × DBP − 0.18 × age − 1.91, respectively.

### 2.3 Diagnostic criteria for DM and DR

The diagnosis of T2DM was made according to the diagnostic criteria of the American Diabetes Association (17). Our study categorized participants as diabetic based on their fasting plasma glucose (FPG), random plasma glucose and HbA1c. The diagnosis and classification of DR was performed following the International Clinical Diabetic Retinopathy Severity Scales (18). Patients were determined to have DR by the presence of any evidence of DR in either eye. Next, participants were further divided into three groups—no diabetic retinopathy (NDR), NPDR and PDR.

### 2.4 Statistical analyses

Continuous variables were described by mean ± standard deviation (SD) when they followed a normal distribution and median (interquartile range) if otherwise. Categorical variables were displayed as numbers with percentages (%). One-way ANOVA was applied to compare differences among three groups for normally distributed quantitative variables; the Kruskal-Wallis test was used for skewed distributions of quantitative variables; for group comparisons of categorical variables, the chi-square test was performed. We performed multivariable logistic regression with PDR as the reference group to compare the associations of target parameters with NDR (vs. PDR) and NPDR (vs. PDR), respectively. For the tertile analyses, MOPP, SBP, and PP were categorized into tertiles and entered into the logistic regression models. For the nonlinear and threshold analyses, PDR was modeled as a binary outcome, with the NDR and NPDR groups combined as the non-PDR group. Two-piecewise logistic regression was then used to examine nonlinear associations and identify potential inflection points. All statistical analyses were carried out using R 4.3.0 statistical software (http://www.R-project.org). P < 0.05 was considered statistically significant.

## 3 Results

### 3.1 Baseline characteristics of the study population

A total of 1,323 patients with T2DM were enrolled in this study, including 551 males (41.7%) and 772 females (58.3%). The median age was 63.0 years (Q1–Q3, 58.0–67.0), and the median duration of DM was 8.0 years (Q1–Q3, 2.0–15.0). Median SBP, DBP, and PP were 144.0 (Q1-Q3 = 131.3, 157.5) mmHg, 84.5 (Q1-Q3 = 78.0, 91.5) mmHg, and 59.5 (Q1-Q3 = 48.0, 71.3) mmHg, respectively. The mean MOPP was 53.0 ± 8.5 mmHg. **Table 1** compared the baseline characteristics of the NDR, NPDR and PDR groups. Sex, age, BMI, HbA1c, insulin use, DM duration, MOPP, SBP, PP, IOP, ALT, AST, TG, UA and CRE differed significantly among the three groups (p<0.05). There were no statistically significant differences in DBP, TC and CSFP.

**Table 1:** Baseline characteristics of the participants.

Variables	Total (n = 1323)	NDR (n = 866)	NPDR (n = 239)	PDR (n = 218)	Statistic	*P*
Age, M (Q_1_, Q_3_)	63.00 (58.00, 67.00)	63.00 (59.00,68.00)	62.00 (58.00,66.00)	59.00 (55.00,65.00)	χ^2^=48.39#	<.001
Sex, n (%)					χ^2^=8.39	0.015
Male	551 (41.65)	340 (39.26)	102 (42.68)	109 (50.00)		
Female	772 (58.35)	526 (60.74)	137 (57.32)	109 (50.00)		
Duration of DM, M (Q_1_, Q_3_)	8.00 (2.00, 15.00)	5.00 (2.00,10.00)	10.00 (5.00,18.00)	15.00 (9.00,20.00)	χ^2^=170.08#	<.001
BMI, M (Q_1_, Q_3_)	26.64 (24.30, 28.98)	26.86 (24.52,29.09)	26.76 (24.74,29.07)	24.77 (22.86,27.34)	χ^2^=37.99#	<.001
Insulin use, n (%)					χ^2^=61.65	<.001
No	934 (77.00)	652 (83.16)	123 (58.02)	159 (73.27)		
Yes	279 (23.00)	132 (16.84)	89 (41.98)	58 (26.73)		
SBP, M (Q_1_, Q_3_)	144.00 (131.25, 157.50)	142.00 (130.50,155.50)	144.25 (130.25,157.12)	150.00 (140.00,165.25)	χ^2^=33.01#	<.001
DBP, M (Q_1_, Q_3_)	84.50 (78.00, 91.50)	84.00 (77.50,91.50)	84.00 (77.38,91.12)	87.00 (79.88,92.00)	χ^2^=4.61#	0.100
IOP, M (Q_1_, Q_3_)	17.00 (14.23-19.00)	17.00 (15.00-20.00)	17.00 (14.00-19.00)	15.35 (13.00-17.00)	χ^2^=66.67#	<.001
CSFP, M (Q_1_, Q_3_)	12.11 (10.26, 13.95)	12.10 (10.16,13.97)	12.14 (10.49,13.97)	12.17 (10.38,13.67)	χ^2^=0.76#	0.683
PP, M (Q_1_, Q_3_)	59.50 (48.00, 71.25)	57.50 (47.00,69.50)	59.25 (47.38,72.00)	64.50 (54.00,79.00)	χ^2^=28.31#	<.001
MOPP, Mean ± SD	53.03 ± 8.45	52.05 ± 8.15	53.24 ± 8.79	56.69 ± 8.26	F=26.83	<.001
HbA1c, M (Q_1_, Q_3_)	7.10 (6.50, 8.10)	7.00 (6.50,7.80)	7.60 (6.70,8.60)	7.30 (6.70,8.30)	χ^2^=38.76#	<.001
ALT, M (Q_1_, Q_3_)	20.00 (15.00, 27.00)	20.00 (15.00,28.00)	19.00 (14.00,25.00)	18.50 (14.00,25.00)	χ^2^=7.96#	0.019
AST, M (Q_1_, Q_3_)	19.00 (16.00, 23.00)	19.00 (16.00,23.00)	17.00 (15.00,22.00)	19.00 (16.00,23.00)	χ^2^=13.98#	<.001
TC, M (Q_1_, Q_3_)	5.20 (4.40, 5.90)	5.20 (4.40,5.90)	5.10 (4.32,5.90)	5.18 (4.18,5.77)	χ^2^=1.79#	0.409
TG, M (Q_1_, Q_3_)	1.57 (1.14, 2.19)	1.62 (1.18,2.20)	1.48 (1.04,2.17)	1.48 (1.12,2.17)	χ^2^=6.80#	0.033
UA, M (Q_1_, Q_3_)	309.00 (259.00, 359.50)	314.00 (265.00,362.75)	302.00 (242.00,353.50)	296.00 (238.25,350.00)	χ^2^=10.79#	0.005
CRE, M (Q_1_, Q_3_)	60.10 (50.00, 72.00)	59.00 (48.40,69.15)	57.20 (48.05,68.15)	71.70 (60.97,91.90)	χ^2^=119.82#	<.001

F, ANOVA; #, Kruskal–Wallis test; χ^2^, Chi-square test.

NDR, no diabetic retinopathy; NPDR, non-proliferative diabetic retinopathy; PDR, proliferative diabetic retinopathy; SD, standard deviation; M, Median; Q1, 1st Quartile; Q3, 3rd Quartile; DM, diabetes mellitus; BMI, body mass index; SBP, systolic blood pressure; DBP, diastolic blood pressure; IOP, intraocular pressure; CSFP, cerebrospinal fluid pressure; PP, pulse pressure; MOPP, mean ocular perfusion pressure; HbA1c, hemoglobin A1c; ALT, alanine aminotransferase; AST, aspartate aminotransferase; TC, total cholesterol; TG, triglyceride; UA, uric acid; CRE, creatinine.

### 3.2 Association between SBP, MOPP, PP and PDR

**Table 2** presents adjusted ORs from multivariable logistic regression models with PDR as the reference category, comparing NDR and NPDR with PDR. With PDR as the reference category, higher MOPP, SBP, and PP were each associated with PDR when compared with NDR and NPDR. The adjusted ORs for NDR and NPDR relative to PDR were 0.92 (95% CI, 0.89–0.95) and 0.94 (95% CI, 0.91–0.98) for MOPP, 0.96 (95% CI, 0.95–0.96) and 0.97 (95% CI, 0.96–0.99) for SBP, and 0.96 (95% CI, 0.95–0.98) and 0.97 (95% CI, 0.96–0.99) for PP, respectively.

**Table 2:** Adjusted ORs from multivariable logistic regression models, with the PDR group as the reference category (n = 218).

Variables	Model 1	Model 2	Model 3
NDR (n = 866)			
Sex	0.93 (0.55-1.56)	0.81 (0.48-1.36)	1.01 (0.60-1.70)
Age	1.21 (1.16-1.26) **	1.18 (1.14-1.23) **	1.20 (1.17-1.27) **
Insulin use	1.56 (0.88-2.76)	1.61 (0.91-2.84)	1.65 (0.94-2.90)
Duration of DM	0.86 (0.83-0.89) **	0.85 (0.82-0.88) **	0.87 (0.84-0.89) **
BMI	1.17 (1.09-1.26) **	1.15 (1.06-1.24) **	1.16 (1.08-1.24) **
ALT	1.00 (0.98-1.03)	1.00 (0.98-1.03)	0.99 (0.98-1.02)
AST	1.01 (0.96-1.02)	1.01 (0.96-1.06)	1.02 (0.97-1.07)
HbA1c	0.81 (0.68-0.96) *	0.82 (0.69-0.97) *	0.79 (0.67-0.94) *
TG	1.23 (0.98-1.53)	1.22 (0.99-1.51)	1.19 (0.96-1.48)
UA	1.00 (0.99-1.01)	1.00 (1.00-1.01) *	1.00 (0.99-1.01)
CRE	0.96 (0.94-0.97) **	0.96 (0.94-0.98) **	0.96 (0.94-0.97) **
IOP	1.20 (1.12-1.29) **	1.11 (1.03-1.19) *	1.19 (1.11-1.27) **
SBP	0.96 (0.95-0.96) **	NA	NA
MOPP	NA	0.92 (0.89-0.95) **	NA
PP	NA	NA	0.96 (0.95-0.98) **
NPDR (n = 239)			
Sex	0.70 (0.39-1.25)	0.64 (0.36-1.13)	0.74 (0.42-1.33)
Age	1.12 (1.07-1.17) **	1.10 (1.05-1.15)**	1.13 (1.08-1.18) **
Insulin use	3.31 (1.82-6.02) **	3.41 (1.88-6.19) **	3.41 (1.88-6.19) **
Duration of DM	0.92 (0.89-0.96) **	0.92 (0.89-0.95) **	0.92 (0.90-0.96) **
BMI	1.19 (1.10-1.28) **	1.18 (1.09-1.27) **	1.18 (1.09-1.27) **
ALT	0.99 (0.96-1.02)	0.99 (0.96-1.02)	0.99 (0.96-1.02)
AST	1.01 (0.96-1.06)	1.01 (0.96-1.07)	1.01 (0.96-1.07)
HbA1c	0.98 (0.82-1.18)	0.99 (0.83-1.20)	0.97 (0.81-1.16)
TG	1.25 (0.99-1.57)	1.24 (0.99-1.56)	1.22 (0.98-1.52)
UA	1.00 (0.99-1.004)	1.00 (0.99-1.01)	1.00 (0.99-1.04)
CRE	0.95 (0.94-0.97) **	0.95 (0.94-0.97) **	0.95 (0.94-0.97) **
IOP	1.12 (1.04-1.21) *	1.06 (0.98-1.14)	1.11 (1.03-1.20) **
SBP	0.97 (0.96-0.99) **	NA	NA
MOPP	NA	0.94 (0.91-0.98) **	NA
PP	NA	NA	0.97 (0.96-0.99) *

NDR, no diabetic retinopathy; NPDR, non-proliferative diabetic retinopathy; PDR, proliferative diabetic retinopathy; OR, odds ratio; CI, confidence interval; SBP, systolic blood pressure; MOPP, mean ocular perfusion pressure; PP, pulse pressure; BMI, body mass index; IOP, intraocular pressure; DM, diabetes mellitus; ALT, alanine aminotransferase; AST, aspartate aminotransferase; HbA1c, glycated hemoglobin; TG, triglycerides; UA, uric acid; CRE, creatinine; NA, not applicable. Model 1=adjusted for Age, Sex, BMI, IOP, Insulin use, Duration of DM, ALT, AST, HbA1c, TG, UA, CRE, SBP. Model 2=Model 1 plus additional adjustment for MOPP and minus adjustment for SBP. Model 3=Model 1 plus additional adjustment for PP and minus adjustment for SBP.*p<0.05, **p<0.001.

We categorized the patients into tertiles according to SBP (≤136, 136-152, ≥152 mmHg), MOPP (≤48, 48-56, ≥56 mmHg), and PP (≤51, 51-67, ≥67 mmHg). **Table 3** showed the ORs from the multivariable logistic analysis for the stratified tertiles of SBP, MOPP, and PP. A significant inverse trend was observed across MOPP tertiles (P for trend <0.01). Compared with the highest tertile, the lowest MOPP tertile was associated with higher odds of NDR (OR, 5.24; 95% CI, 2.73–10.08) and NPDR (OR, 3.53; 95% CI, 1.74–7.16) relative to PDR. The lowest SBP tertile was also associated with higher odds of NDR relative to PDR (OR, 4.97; 95% CI, 2.28–9.19). For PP, the lowest tertile was associated with higher odds of both NDR (OR, 4.05; 95% CI, 2.18–7.53) and NPDR (OR, 3.27; 95% CI, 1.68–6.37) relative to PDR.

**Table 3:** Adjusted associations of SBP, MOPP, and PP tertiles with DR severity, with PDR as the reference group.

Variables	OR (95%CI)	P value	P for trend
NDR			
SBP ^a^			<.001
Bottom tertile (≤136)	4.966(2.284-9.188)	<.001	
Middle tertile (136–152)	2.418(1.394-4.191)	0.002	
Top tertile (≥152)	Reference		
MOPP ^b^			<.001
Bottom tertile (≤48)	5.242(2.727-10.076)	<.001	
Middle tertile (48–56)	2.122(1.249-3.606)	0.005	
Top tertile (≥56)	Reference		
PP ^c^			<.001
Bottom tertile (≤51)	4.054(2.181-7.534)	<.001	
Middle tertile (51–67)	1.966(1.136-3.403)	0.016	
Top tertile (≥67)	Reference		
NPDR			
SBP ^a^			<.001
Bottom tertile (≤136)	3.600(1.854-6.991)	<.001	
Middle tertile (136–152)	1.970(1.074-3.615)	0.029	
Top tertile (≥152)	Reference		
MOPP ^b^			0.001
Bottom tertile (≤48)	3.533(1.743-7.161)	<.001	
Middle tertile (48–56)	1.463(.812-2.638)	0.206	
Top tertile (≥56)	Reference		
PP ^c^			0.001
Bottom tertile (≤51)	3.269(1.677-6.371)	0.001	
Middle tertile (51–67)	1.328(.718-2.456)	0.366	
Top tertile (≥67)	Reference		

NDR, no diabetic retinopathy; NPDR, non-proliferative diabetic retinopathy; PDR, proliferative diabetic retinopathy; OR, odds ratio; CI, confidence interval; SBP, systolic blood pressure; MOPP, mean ocular perfusion pressure; PP, pulse pressure. The cutoffs were based on tertiles of the population distribution (33.3rd and 66.7th percentiles). a=adjusted for Age, Sex, BMI, IOP, Insulin use, Duration of DM, ALT, AST, HbA1c, TG, UA, CRE, SBP. b=a plus additional adjustment for MOPP and minus adjustment for SBP. c=a plus additional adjustment for PP and minus adjustment for SBP.

### 3.3 Non-linear regression and threshold effect analysis

Exploratory nonlinear and threshold analyses were then performed to assess whether the associations with PDR departed from linearity. Threshold effect analysis was shown in **Figure 2**. PP showed a non-linear relationship with PDR (P for likelihood test=0.046), while no non-linear relationship was observed for SBP (P for likelihood test=0.511) and MOPP (P for likelihood test=0.368). The inflection point of the PP was 38.5 mmHg in **Table 4**. Segmented logistic regression analysis determined that when PP ≥ 38.5 mmHg, the odds of PDR were 4% higher for each 1-mmHg increase in PP (OR, 1.04; 95% CI, 1.03–1.06). In contrast, when PP < 38.5 mmHg, the association between PP and PDR was not statistically significant (OR, 0.92, 95% CI, 0.77-1.09, P = 0.309), indicating that the negative slope in this segment lacked statistical support.

**Figure 2:**
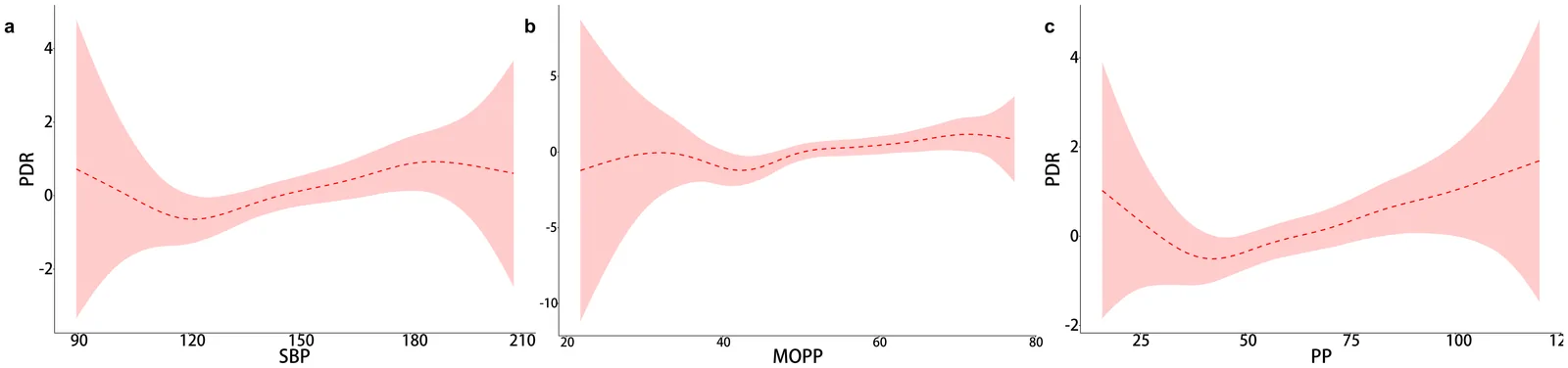
The nonlinear relationship between SBP **(a)**, MOPP **(b)**, PP **(c)** and PDR. A smooth curve fitting between variables is represented by the red dashed line. The 95% confidence interval of the fit is indicated by the red shaded areas.

**Table 4:** Exploratory analysis of nonlinear associations of SBP, MOPP, and PP with PDR using two-piecewise linear regression.

Variables	Outcome	Effect	*P*
SBP ^a^	Model 1 Fitting model by standard linear regression	1.03 (1.02 - 1.05)	<.001
	Model 2 Fitting model by two-piecewise linear regression		
	Inflection point	158.999	
	<158.999	1.04 (1.02 - 1.06)	<.001
	≥158.999	1.03 (0.95 - 1.12)	0.449
	P for likelihood test		0.511
MOPP ^b^	Model 1 Fitting model by standard linear regression	1.08 (1.05 - 1.11)	<.001
	Model 2 Fitting model by two-piecewise linear regression		
	Inflection point	41.444	
	<41.444	14188.53 (0.00 - Inf)	0.999
	≥41.444	1.09 (1.05 - 1.12)	<.001
	P for likelihood test		0.368
PP ^c^	Model 1 Fitting model by standard linear regression	1.03 (1.02 - 1.05)	<.001
	Model 2 Fitting model by two-piecewise linear regression		
	Inflection point	38.5	
	<38.5	0.92 (0.77 -1.09)	0.309
	≥38.5	1.04 (1.03 - 1.06)	<.001
	P for likelihood test		0.046

OR, odds ratio; CI, confidence interval; SBP, systolic blood pressure; MOPP, mean ocular perfusion pressure; PP, pulse pressure; PDR, proliferative diabetic retinopathy. a=adjusted for Age, Sex, BMI, IOP, Insulin use, Duration of DM, ALT, AST, HbA1c, TG, UA, CRE, SBP. b=a plus additional adjustment for MOPP and minus adjustment for SBP. c=a plus additional adjustment for PP and minus adjustment for SBP.

**Table 5**, **Figure 3** showed that higher IOP was inversely associated with PDR when IOP was ≤ 17.5 mmHg (OR, 0.79, 95% CI, 0.70-0.89), and this association disappeared when IOP was > 17.5 mmHg. The two-piecewise linear regression model was better than the standard linear regression model for fitting the association between IOP and PDR.

**Table 5:** Exploratory analysis of the nonlinear association between IOP and PDR using two-piecewise linear regression.

Outcome	Effect	*P*
Model 1 Fitting model by standard linear regression	0.87 (0.82 - 0.93)	<.001
Model 2 Fitting model by two-piecewise linear regression		
Inflection point	17.48	
<17.48	0.79 (0.70 - 0.89)	<.001
≥17.48	1.04 (0.94 - 1.16)	0.455
P for likelihood test		<.001

OR, odds ratio; CI, confidence interval; IOP, intraocular pressure; PDR, proliferative diabetic retinopathy. Adjusted for Age, Sex, BMI, Insulin use, Duration of DM, ALT, AST, HbA1c, TG, UA, CRE.

**Figure 3:**
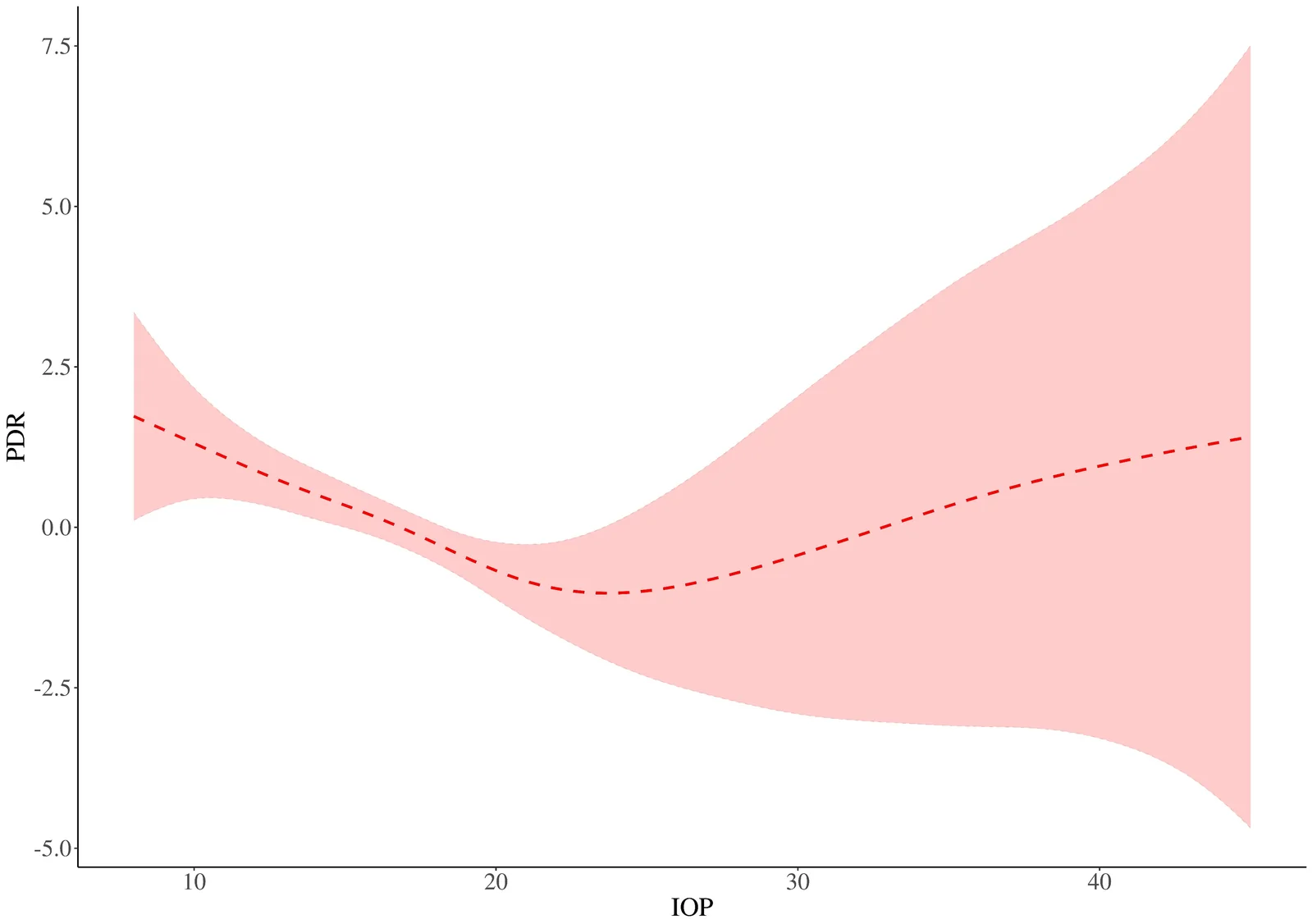
The Nonlinear relationship between IOP and PDR. A smooth curve fitting between variables is represented by the red dashed line. The 95% confidence interval of the fit is indicated by the red shaded areas.

## 4 Discussion

In this retrospective cross-sectional study, we examined the associations of MOPP, SBP, PP, and IOP with DR severity. After adjustment for covariates, higher MOPP was associated with PDR when compared with both NDR and NPDR. No evidence of a nonlinear association between MOPP and PDR was observed.

As early as the 1990s, a study revealed that retinal blood flow was notably elevated in DR patients, in comparison to both non-diabetic individuals and diabetic patients without retinopathy. This study further demonstrated an association between hyperperfusion and the severity of retinopathy in the retinal circulation (6). The elevated retinal perfusion pressure induces hemodynamic stresses (circumferential and shear stress), directly damaging vascular endothelial cells and disrupting the blood-retinal barrier. This cascade increases capillary permeability, driving fluid exudation (per Starling’s principle) and subsequent retinal edema with hard exudate formation (18). Chronic microvascular injury manifests as microaneurysms, hemorrhages, and plasma leakage. Sustained chronic damage promotes capillary occlusion. Progressive ischemia upregulates angiogenic factors, transitioning to PDR (19).

Originally recognized as the driving force of human ocular blood circulation in the eye, MOPP was later proposed as an indicator of ocular perfusion status, as it combines IOP, SBP, and DBP (20, 21). Higher MOPP leads to an increase in retinal blood flow, subsequently elevating the hydrostatic pressure of retinal capillaries and enhancing capillary leakage.

To date, conclusions on the association between MOPP and DR remain inconsistent (7–9, 22–24). Results from the Fushun Diabetic Retinopathy Cohort Study (FS-DIRECT) indicate a significant association between each 1 mmHg increase in MOPP and any type of DR, particularly NPDR, non-sight threatening diabetic retinopathy (non-STDR), and macular edema in northeastern Chinese T2DM patients (8). Longitudinal data collected by FS-DIRECT revealed that elevated MOPP was correlated with the incidence of DR and was borderline negatively correlated with the regression of DR. However, no association was found between MOPP and the development of DR (7). Moreover, data from a four-year population-based cohort study found that MOPP exhibited a significant association with the incidence of DR only in young-onset patients (diagnosed before the age of 30), whereas no such association was observed in older-onset patients (diagnosed after the age of 30). Notably, for every 10 mmHg increase in MOPP, the odds of developing DR increased by approximately 113% (22). Multivariate analysis showed no association between MOPP and DR among diabetic patients in southern India, according to a report from the Sankara Nethralaya Diabetic Retinopathy Epidemiology and Molecular Genetics Study (SN-DREAMS) (23). Kumari et al. (24) similarly observed no association between MOPP and the incidence and progression of DR among Indian immigrants residing in Singapore. These conflicting findings may be attributed to discrepancies in baseline data and study design. In the present study, although the MOPP curve showed some fluctuation at lower values, formal model comparison did not support a better fit of the two-piecewise linear model than the standard linear model (P for likelihood test = 0.368), supporting an overall linear association between MOPP and PDR.

As a significant component of the MOPP formula, BP has long been identified as a major risk factor for the development of PDR (25, 26). At the same time, we also concentrated on the role of PP in PDR. Defined as the difference between SBP and DBP, PP has not been adequately investigated in DR progression, but in a study by Ishihara et al. (27), PP was proved to be a notable factor together with SBP. PP is associated with severe eye disease and relies on the elasticity of the arterial wall (13). In the present study, higher SBP and PP were associated with PDR when compared with both NDR and NPDR, whereas DBP did not differ significantly among the three groups. These findings are broadly consistent with previous reports (28, 29). In the exploratory nonlinear analysis, PP was positively associated with PDR at values ≥38.5 mmHg, whereas the association below this value was not statistically significant. One possible explanation is that greater pulsatile pressure may expose the retinal microvasculature to increased cyclic hemodynamic stress. In diabetes, impaired vascular autoregulation and endothelial dysfunction may increase susceptibility to such pulsatile stress. However, the estimated inflection point was data-derived and should not be interpreted as a clinical threshold. Confirmation in independent studies is needed.

Earlier clinical observations have also reported lower IOP in eyes with PDR, with IOP decreasing in parallel with retinopathy severity in eyes without glaucoma (30). Although the direction of this association remains uncertain, these findings suggest that lower IOP may accompany advanced diabetic retinal disease. At a given blood pressure, lower IOP also corresponds to higher estimated ocular perfusion pressure. In the presence of diabetes-related impairment of retinal vascular autoregulation, this altered hemodynamic environment may be associated with the retinal perfusion abnormalities discussed above. However, the underlying ocular mechanism remains unclear. Our cross-sectional data cannot distinguish whether lower IOP precedes PDR or reflects ocular changes accompanying advanced disease. In addition, the 17.5 mmHg inflection point was data-derived and should not be interpreted as a clinical threshold.

To our knowledge, our study is the first to directly compare the associations of MOPP, SBP, and PP across NDR, NPDR, and PDR groups. It is crucial to acknowledge several limitations inherent in the present study. First, the cross-sectional nature of the study design precludes the determination of causality and temporality. Second, considering the present data were derived from two distinct populations, the study population is somewhat heterogeneous. Third, the enrichment of PDR cases from a surgical cohort may over-represent more severe cases, potentially affecting generalizability. Fourth, the calculation of MOPP using the theoretical formula may not accurately reflect the actual physiological state of ocular perfusion. Different results may be obtained from direct measurements of retinal blood flow. Fifth, the study only included Chinese patients over the age of 50, caution is therefore needed when attempting to extrapolate the results to other age groups and ethnic groups. Additional research is warranted to ascertain whether these findings can be generalized to other groups.

## 5 Conclusions

In conclusion, higher MOPP was associated with PDR relative to both NDR and NPDR in this cross-sectional study. These findings suggest that MOPP may provide complementary hemodynamic information in relation to DR severity. However, because MOPP incorporates BP and IOP components and this study was cross-sectional, causal inference and predictive interpretation are not warranted. Longitudinal studies are needed to confirm these findings.

## Data Availability

The data analyzed in this study is subject to the following licenses/restrictions: NA. Requests to access these datasets should be directed to Juping Liu, tydljp@126.com.
